# Prevalence and factors associated with comorbid depressive symptoms among people with low back pain in China: A cross-sectional study

**DOI:** 10.3389/fpsyt.2022.922733

**Published:** 2022-07-25

**Authors:** Chunxia He, Hongxiu Chen, Ling Guo, Lisheng Xu, Qingquan Liu, Jiali Zhang, Xiuying Hu

**Affiliations:** ^1^West China School of Nursing, Sichuan University/Institute for Disaster Management and Reconstruction, and Innovation Center of Nursing Research, West China Hospital, Sichuan University, Chengdu, China; ^2^Nursing Key Laboratory of Sichuan Province, West China Hospital, Sichuan University, Chengdu, China; ^3^Affiliated Hospital of North Sichuan Medical College, Nanchong, China; ^4^Cheng Du Shang Jin Nan Fu Hospital, Chengdu, China

**Keywords:** low back pain, depressive symptoms, comorbidity, prevalence, associated factors

## Abstract

**Background:**

Low back pain is a common medical condition among the general population that is associated with many adverse health effects when comorbid with depressive symptoms. However, little is known about depressive symptoms in the population with low back pain in China. Our study evaluated the prevalence of depressive symptoms and analyzed the factors associated with this condition in the Chinese population with low back pain.

**Methods:**

We conducted a cross-sectional analysis of data from the 2018 China Health and Retirement Longitudinal Study. We collected low back pain information for each participant and identified depressive symptoms using the brief version of the Center for Epidemiologic Studies Depression Scale. A wide range of sociodemographic and health-related characteristics of the subjects were extracted. We measured the prevalence of depressive symptoms comorbid with low back pain and analyzed the associated factors by multiple logistic regression.

**Results:**

A total of 5,779 respondents aged 45 and over with low back pain formed the sample, 41.8% of whom reported depressive symptoms. Multiple logistic regression analysis indicated greater vulnerability to depressive symptoms among females (OR = 1.41, 95% CI, 1.16–1.73), relatively younger persons (60–74 years: OR = 0.72, 95% CI, 0.63–0.83; ≥ 75 years: OR = 0.62, 95% CI, 0.49–0.79, reference: 45–59 years), those from the central and western regions (central: OR = 1.39, 95% CI, 1.18–1.64; western: OR = 1.56, 95% CI, 1.33–1.83), participants with extremely short sleep duration (OR = 2.74, 95% CI, 2.33–3.23), those with poor self-perceived health status (OR = 2.91, 95% CI, 2.34–3.63,), multisite pain (OR = 1.54, 95% CI, 1.20–1.98) and disability in activities of daily living (Basic: OR = 1.70, 95% CI, 1.47–1.98; Instrumental: OR = 1.95, 95% CI, 1.70–2.24).

**Conclusion:**

Depressive symptoms were highly prevalent in the Chinese population ≥ 45 years with low back pain. More attention should be paid to the individuals at high-risk confirmed by this study to facilitate early identification and intervention against depressive symptoms.

## Introduction

Low back pain (LBP) refers to pain or discomfort that originates between the lower rib margins and the buttock creases and may be accompanied by referred leg pain (1). It is an urgent public health concern because of its high prevalence and role as a cause of disability worldwide. A systematic review of 165 publications estimated that the point prevalence of LBP reaches 11.9% and the monthly prevalence is 23.2% (2). According to the Global Burden of Disease study, LBP contributed to 64.9 million disability-adjusted life years in 2017, an increase of 47.5% since 1990 (3). In China, the LBP situation is similarly problematic; it is the second-leading cause of disability, inflicting substantial health and economic burdens on society (4, 5).

Individuals with LBP have an increased likelihood of experiencing psychiatric comorbidity than do the general population (6, 7), of which depressive symptoms are the most common, with a reported prevalence of 4–50% (8, 9). Previous studies confirmed that the coexistence of depressive symptoms significantly predicts persistent disabling LBP (positive LR, 2.2) (10). Furthermore, the possible adverse effects of comorbid depressive symptoms have been explored in studies in the United States (11), Denmark (12), and Australia (13). These studies found that comorbid depressive symptoms not only contribute to the deterioration of pain and body function but also increase health care resource utilization. A systematic review also found markedly reduced psychosocial and physical quality of life among such patients (14). Collectively, the management of depressive symptoms in people with LBP is crucial in clinical practice. Understanding the factors associated with depressive symptoms can provide relevant evidence to guide the development of mental healthcare strategies in the future.

In recent years, many studies have been conducted on the factors that influence comorbid depressive symptoms. However, some of these reports only provided univariate analyses and did not assess possible synergistic or confounding effects among factors (15, 16); others explored a broader age range of participants ( ≥ 18 years) (17, 18), while the prevalence of LBP shows a curvilinear distribution with age, with the greatest prevalence in people 40–80 years of age (2), caution is warranted in generalizing the existing findings to older age groups. Additionally, to the best of our knowledge, individuals with LBP in China have received relatively little research attention, and the most relevant prior study used convenience sampling to select participants from only two hospitals (19). Given these limitations, we conducted a more extensive nationwide survey of the epidemiology of comorbid depressive symptoms among middle-aged and older Chinese people with LBP and further explored its associated characteristics.

## Methods

### Sample

We employed cross-sectional data collected from wave 4 of the China Health and Retirement Longitudinal Study (CHARLS) in 2018. CHARLS is a community-based longitudinal project hosted by the National School of Development at Peking University. Participants aged 45 or above were eligible for the study, which used a stratified (by GDP per capita in urban and rural counties) multistage (county/district-village/community households) probability sampling design. Demographic, household, and health-related information was collected utilizing the Computer-Assisted Personal Interview (CAPI) system during one-to-one visits. The median interview time was 94.33 min. The CHARLS national baseline survey spanned 28 provinces, 150 cities/districts, 450 urban/rural communities, and approximately 17,000 people in 10,000 households. These individuals were followed up every 2 to 3 years to capture updated information. The project also obtained a high participation rate, with a response rate of 80.5% for the baseline and over 86% for the follow-up sample. It provides a high-quality micro database to serve the research needs related to China's rapidly aging population. Detailed information about the cohort can be found elsewhere (20). CHARLS received ethical approval from Peking University's Institutional Review Board (Approval No. IRB00001052-11015); all respondents provided informed consent.

**Figure 1:**
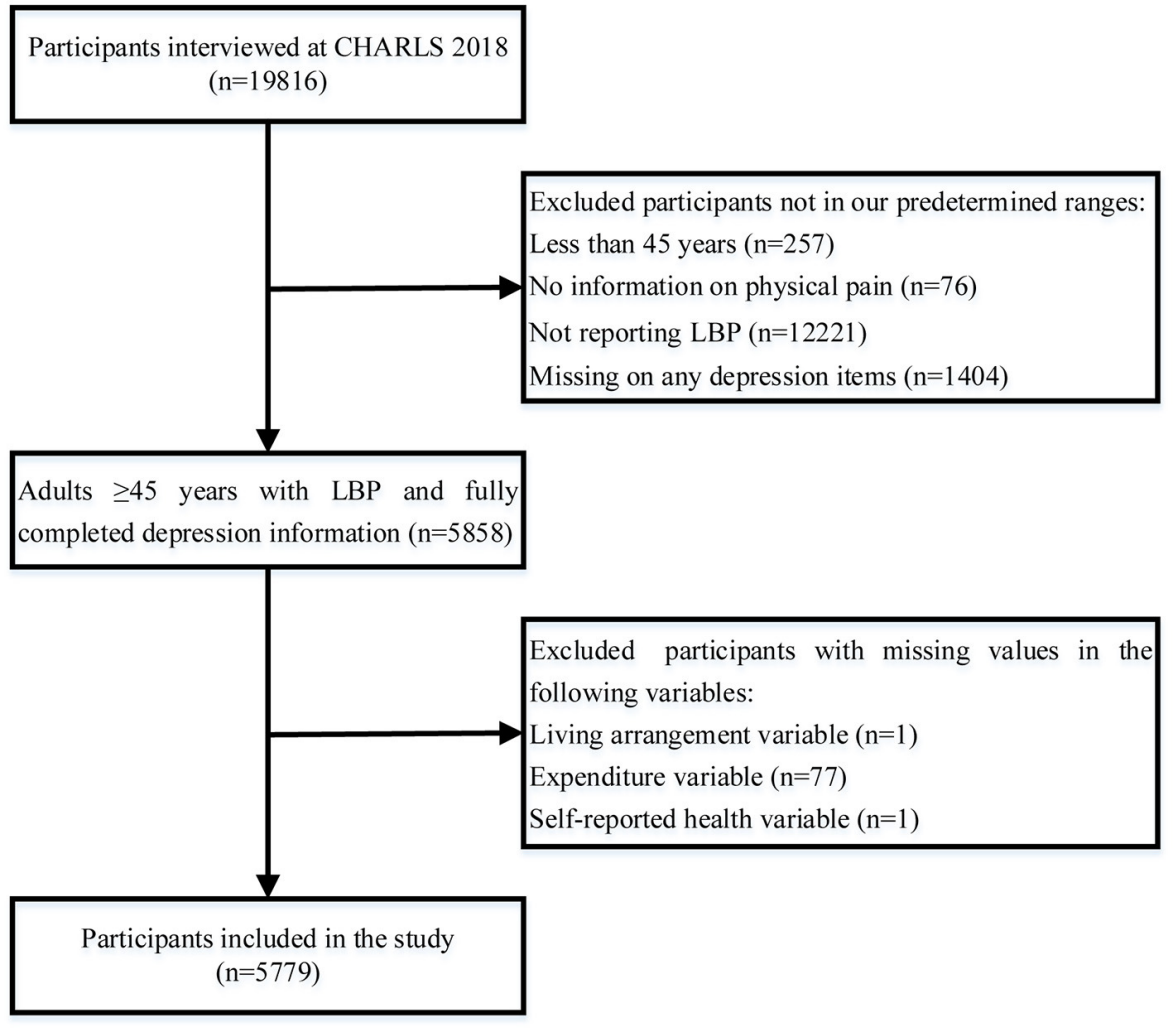
Flowchart for screening study subjects. CHARLS, China Health and Retirement Longitudinal Study; LBP, Low back pain.

A total of 19,816 respondents were collected for CHARLS 2018. Based on the purpose of our study, subjects were excluded from the analysis if younger than 45 years, if they provided no information about physical pain, if they did not report LBP, or if any depression scale items were missing. Additionally, we removed those participants with missing values on independent variables among the remaining respondents. The final sample consisted of 5,779 participants. The screening flow is outlined in Figure 1.

### Identification of LBP

In the questionnaire, respondents were asked if they suffered from any physical pain often. If affirmative, they were instructed to list the body parts from which they felt pain. Locations of the pain included the head, shoulders, arms, wrists, fingers, chest, stomach, back, lower back, buttocks, legs, knees, ankles, toes, neck, and others. Respondents were considered to have LBP if they marked the lower back.

### Assessment of depressive symptoms

In the CHARLS, the 10-item version of the Center for Epidemiological Studies Depression Scale (CESD-10), a modification of the CESD-20 scale (21), was applied to measure the depressive symptoms of respondents during the previous week. The shortened scale comprises eight negative items and two positive items. The former includes feeling depressed, scared and lonely, bothered by matters, hard to pay attention, having difficulty with daily chores, not sleeping well, and not being able to cope with life as a whole. In contrast, the latter includes feeling hopeful about the future and feeling glad. The frequency with which respondents experience the feelings listed is rated on four levels: “rarely or none (< 1 day),” “some or a few (1–2 days),” “occasional or moderate (3–4 days),” and “most or all (5–7 days).” Item scores range from 0 to 3 points, whereas positive items are reverse scored. Total CESD-10 scores range between 0 and 30. Scores of 12 or greater were regarded as denoting the presence of comorbid depressive symptoms (22, 23). Previous studies have demonstrated that this scale is valid and reliable (24); Cronbach's alpha was 0.80 in this study.

### Covariates

Depressive symptoms among individuals with LBP are subject to interactions of multiple complex factors. Based on an extensive literature review, we considered selected sociodemographic variables in our analysis, namely sex, age (45–59, 60–74, ≥ 75), region [classified by the National Bureau of Statistics of China consistent with socioeconomic development conditions (25)], current residence (urban or rural), ethnicity (Han or ethnic minorities), marital status (separated/divorced/widowed/unmarried, married/cohabitating), and health insurance (public and private health insurance coverage: no, yes). Furthermore, we assessed the following socioeconomic status characteristics of the respondents: education (primary or lower, middle, high or vocational, college or higher), type of work (currently not working or never worked, agricultural, and non-agricultural), and monthly household expenditure per capita (≤ 500 or > 500 CNY, defined based on the median).

Studies have also shown that health-related factors are strong predictors of depressive symptoms (26–29). The variables used in this study to reflect health status addressed sleep, physical activity, social activities, smoking, alcohol consumption, self-reported health (SRH), number of chronic conditions, multisite pain, and activities of daily living (ADL). We recorded the self-reported duration of nighttime sleep (within the past month) as a measure of sleep habits. Participants were assigned to one of four nighttime sleep categories based on existing epidemiological literature (30): extremely short sleep (< 5 h/night), short sleep (5–7 h/night), normal sleep (7–9 h/night), and excessive sleep (≥ 9 h/night). Regarding physical activity, participants were asked about intensity (vigorous, moderate, mild), daily duration (0, 10–30 min, 30–120 min, 120–240 min, ≥ 240 min), and weekly frequency (1–7 days). To complete the assessment, we used the median duration of each intensity level as a proxy for the duration of each group (“240 min” for the “ ≥ 240 min” group) and subsequently generated the total amount of physical activity from the duration and weekly frequency. According to WHO recommendations (31), “sufficient physical activity” refers to over 150 min of moderate activity or over 75 min of vigorous activity per week, and “insufficient physical activity” denotes less activity than that. Social activities were coded as “yes” or “no” by asking respondents whether they were involved in certain activities, such as providing help to others and engaging in community organizations. The data on smoking and alcohol consumption were taken directly from the database and categorized into three groups: never, former, and current. Moreover, each participant used a scale of 1–5 to report their current overall health state, with 1 and 5 denoting excellent and very poor respectively. These ratings were then re-stratified into three groups (good, fair, and poor). The number of chronic conditions was measured by the self-reported diagnosis of the following diseases: hypertension, dyslipidemia, diabetes, cancer, chronic pulmonary disease, liver disease, heart disease, stroke, kidney disease, digestive illness, arthritis, and asthma. The analysis did not include psychiatric and memory-related disorders because of the potential for recall bias. During the interview, multiple pains were determined in case the respondents reported pain in other sites besides the lower back. ADLs were assessed, comprising basic activities of daily living (BADL) and instrumental activities of daily living (IADL). The former involved clothing, showering, taking meals, toileting, going to and from bed, and bowel and bladder function; the latter involved housekeeping, preparing meals, shopping, using the phone, administering medication, and finances. Each activity was rated as one of four levels: no difficulty, difficult but capable of completion, difficult and requiring assistance, and impossible. In this sample, we counted BADL or IADL disability as present if there was difficulty with any item.

### Statistical analysis

The statistical analysis was performed with IBM SPSS Statistics, version 26.0. We examined the distribution of the data using descriptive statistics, expressing categorical variables as percentages. Pearson's Chi-Square test was utilized to assess the significance of differences between groups with and without depressive symptoms. Multicollinearity among each variable was not detected using the variance inflation factor (VIF) (MAX VIF = 2.42). Sociodemographic and health-related variables that were significant in univariate analyses (*p* ≤ 0.05) entered a multivariable logistic regression model. We identified the most parsimonious and best-fitting model using a stepwise backward procedure that considered the likelihood ratio. In the final model, we set the threshold for significance of *p*-values at 0.005 because of the effect of the large sample on power and statistical significance (32).

## Results

### Prevalence of depressive symptoms

As shown in Figure 1, a total of 19,483 individuals aged 45 years and over provided physical pain data, of whom 37.3% (*n* = 7,262) reported that they were troubled with LBP. After the screening process, 5,779 participants were enrolled in the estimation of the prevalence of depressive symptoms in people with LBP. The average age was 61.0 years (SD = 9.2), 61.4% of the sample were female, and the prevalence of depressive symptoms was 41.8%.

### Sociodemographic factors associated with depressive symptoms

A comparison of the groups with and without depressive symptoms in the LBP population in terms of sociodemographic characteristics is provided in Table 1. In the univariate analysis, sex, age, region, current residence, marital status, living arrangement, health insurance, education level, type of work, and monthly household expenditure per capita statistically differed between groups. Only sex, age, and region were identified as independent factors associated with depressive symptoms after multivariate adjustment. A higher prevalence was found in females, with the odds being 1.41 times (OR = 1.41, 95% CI, 1.16–1.73) than males, as presented in **Table 3**. The older respondents with LBP tended to have fewer depressive symptoms compared to the middle-aged group (60–74 years: OR = 0.72, 95% CI, 0.63–0.83; ≥ 75 years: OR = 0.62, 95% CI, 0.49–0.79). Residents in the central and western regions owned an odds of 1.39 times (OR = 1.39, 95% CI, 1.18–1.64) and 1.56 times (OR = 1.56, 95% CI, 1.33–1.83) of that in the eastern region, respectively.

**Table 1:** Sociodemographic characteristics of the LBP population by the presence of depressive symptoms (*n* = 5,779).

**Variable**		**Depressive symptoms**		***p*** **-value**
		**No**	**Yes**	
Sex	Male	1,465 (65.6)	768 (34.4)	<0.001
	Female	1,896 (53.5)	1,650 (46.5)	
Age	45–59	1,619 (60.9)	1,039 (39.1)	<0.001
	60–74	1,470 (56.4)	1,135 (43.6)	
	≥ 75	272 (52.7)	244 (47.3)	
Regions	Eastern	929 (67.4)	449 (32.6)	<0.001
	Central	971 (56.5)	747 (43.5)	
	Western	1,169 (53.1)	1,031 (46.9)	
	Northeastern	292 (60.5)	191 (39.5)	
Current residence	Urban	995 (67.1)	488 (32.9)	<0.001
	Rural	2,366 (55.1)	1,930 (44.9)	
Ethnicity	Minority	304 (57.9)	221 (42.1)	0.901
	Han	3,057 (58.2)	2,197 (41.8)	
Marital status	separated/divorced/ widowed/unmarried	356 (44.7)	441 (55.3)	<0.001
	married/cohabiting	3,005 (60.3)	1,977 (39.7)	
Living arrangement	Living alone	178 (42.6)	240 (57.4)	<0.001
	Living with others	3,183 (59.4)	2,178 (40.6)	
Health insurance	No	65 (48.9)	68 (51.1)	0.028
	Yes	3,296 (58.4)	2,350 (41.6)	
Education	Primary or lower	2,112 (53.3)	1,849 (46.7)	<0.001
	Middle	815 (67.1)	400 (32.9)	
	High or vocational	372 (70.3)	157 (29.7)	
	College or higher	62 (83.8)	12 (16.2)	
Type of work	Currently not working or never worked	1,063 (55.2)	862 (44.8)	<0.001
	Agricultural	1,272 (53.3)	1,116 (46.7)	
	Non-agricultural	1,026 (70.0)	440 (30.0)	
Monthly household expenditure per capita	≤ 500	1,749 (55.1)	1,425 (44.9)	<0.001
	> 500	1,612 (61.9)	993 (38.1)	

### Health-related factors associated with depressive symptoms

Table 2 compares the details between the two groups based on a set of self-reported health-related characteristics. Statistically significant differences were found in the comparison in terms of nighttime sleep duration, physical activity, social activity, smoking, alcohol consumption, SRH, number of chronic conditions, multisite pain, and ADL. The fully adjusted model is as indicated in Table 3. Comorbid depressive symptoms were strongly associated with shorter nighttime sleep duration (OR = 2.74, 95% CI, 2.33–3.23), poor SRH (OR = 2.91, 95% CI, 2.34–3.63), and multisite pain (OR = 1.54, 95% CI,1.20–1.98). Additionally, respondents with BADL disability were at greater risk of depressive symptoms than were persons with no limitations in ADL (OR = 1.70, 95% CI, 1.47–1.98), as were persons with IADL disability (OR = 1.95, 95% CI, 1.70–2.24).

**Table 2:** Health-related characteristics of the LBP population by the presence of depressive symptoms (*n* = 5,779).

**Variable**		**Depressive symptoms**		***p*** **-value**
		**No**	**Yes**	
Duration of nighttime sleep	Extremely short	556 (38.2)	899 (61.8)	<0.001
	Short	1,387 (61.0)	886 (39.0)	
	Normal	1,193 (71.1)	485 (28.9)	
	Excessive	225 (60.3)	148 (39.7)	
Physical activity	Insufficient	1,210 (55.4)	974 (44.6)	0.001
	Sufficient	2,151 (59.8)	1,444 (40.2)	
Social activities	No	1,407 (53.6)	1,217 (46.4)	<0.001
	Yes	1,954 (61.9)	1,201 (38.1)	
Smoking	Never	2,020 (55.7)	1,609 (44.3)	<0.001
	Former	456 (63.2)	266 (36.8)	
	Current	885 (62.0)	543 (38.0)	
Alcohol consumption	Never	2,159 (55.7)	1,720 (44.3)	<0.001
	Former	283 (53.5)	246 (46.5)	
	Current	919 (67.0)	452 (33.0)	
SRH	Good	540 (78.7)	146 (21.3)	<0.001
	Fair	1,927 (66.6)	967 (33.4)	
	Poor	894 (40.7)	1,305 (59.3)	
Number of chronic conditions	0	482 (71.5)	192 (28.5)	<0.001
	1	744 (64.7)	406 (35.3)	
	≥ 2	2,135 (54.0)	1,820 (46.0)	
Multisite pain	No	353 (78.6)	96 (21.4)	<0.001
	Yes	3,008 (56.4)	2,322 (43.6)	
BADL disability	No	2,807 (65.8)	1,461 (34.2)	<0.001
	Yes	554 (36.7)	957 (63.3)	
IADL disability	No	2,626 (68.3)	1,220 (31.7)	<0.001
	Yes	735 (38.0)	1,198 (62.0)	

**Table 3:** Multiple regression analysis of factors associated with comorbid depressive symptoms in LBP.

**Variable**		**Odds ratio**	**95% CI**	***p*** **-value**
Sex	Male	1.0	-	0.001
	Female	1.41	1.16–1.73	
Age	45–59	1.0	-	<0.001
	60–74	0.72	0.63–0.83	
	≥ 75	0.62	0.49–0.79	
Regions	Eastern	1.0	-	<0.001
	Central	1.39	1.18–1.64	
	Western	1.56	1.33–1.83	
Duration of nighttime sleep	Normal	1.0	-	<0.001
	Short	1.54	1.33–1.78	
	Extremely short	2.74	2.33–3.23	
SRH	Good	1.0	-	<0.001
	Fair	1.49	1.21–1.84	
	Poor	2.91	2.34–3.63	
Multisite pain	No	1.0	-	0.001
	Yes	1.54	1.20–1.98	
BADL disability	No	1.0	-	<0.001
	Yes	1.70	1.47–1.98	
IADL disability	No	1.0	-	<0.001
	Yes	1.95	1.70–2.24	

## Discussion

Given the lack of nationally representative information on the status of LBP comorbid with depression within China, this study used data from CHARLS 2018 to assess the prevalence of comorbid depressive symptoms in people ≥ 45 years with LBP and the associated factors. We found that depressive symptoms were common in the Chinese middle-aged and older people with LBP. Further, the cross-sectional analysis suggested that sex, age, region, sleep duration, SRH, multisite pain, and physical functional limitations were all associated with depressive status. Exploring the prevalence and possible causes of depressive symptoms will help develop better prevention and intervention strategies for mental health problems in the population with LBP.

A total of 37.3% of participants reported experiencing LBP, which fell within the range of previously reported community studies for similar age groups (5, 33, 34). Further, we observed that the prevalence of depressive symptoms was 41.8% in this study, which is higher than that reported in Korea (20.3%) (16), Nigeria (20.3%) (18), Japan (16.5%) (15), and another study from China (25.0%) (19). There are two possible reasons for this discrepancy: (a) the participants involved in the current study were 45 years of age and older, an age group with a reported prevalence of depressive symptoms of approximately 20.0%-24.1% in the general population (35–37), which is commonly considered to be at relatively high risk of depression in China; and (b) our evaluation of depressive symptoms was based on the CESD-10 scale, which is not intended as a clinical diagnostic instrument but is widely used for the initial screening of depressive symptoms. These reasons might explain the high prevalence of depressive symptoms in our study. Regardless, this relatively common mental health problem among persons with LBP aged 45 years and older deserves greater attention from medical practitioners, researchers, and policymakers.

In our study, the prevalence of depressive symptoms among females was 1.41 times that of males. This gender-specific difference has been observed in national and international epidemiological studies (38, 39). This greater susceptibility was attributed to the biological and psychosocial characteristics specific to women (40). The risk in older adults was approximately half that of middle-aged adults, which was consonant with the results of other comparable studies (39). This correlation between age and depressive symptoms was elaborated as an “inverted U-shaped” distribution in an adult survey, meaning that young and middle-aged individuals were more prone to suffer from depressive symptoms (41). It should be pointed out, however, that the prevalence of LBP tended to increase gradually with age in our sample after *post–hoc* analysis (35.8%, 38.5%, and 38.0% in 45–59, 60–74, and ≥ 75 age groups, respectively). Contributing to this result might be the “well-being paradox” hypothesis of older adults, which stated that despite higher rates of pain in older age groups, they still outperformed younger groups in terms of mental health due to their adaptive mechanisms (42). For the regions, the central and western regions presented higher prevalence than did the eastern region, primarily because of the uneven economic development and medical resource allocation among areas.

We demonstrated that sleep duration was associated with depressive symptoms; specifically, increased odds of depressive symptoms were present in LBP individuals with shorter sleep duration. Sleep disturbances and depressive symptoms often coexist in individuals with LBP (43). The mesolimbic dopaminergic system has been proposed as an important contributor to the dysregulation present in such disorders (44). A longitudinal cohort study from CHARLS (45) indicated that in the general population, those with short sleep duration (< 5 h) were at a greater risk of depressive episodes (OR = 1.69, 95% CI, 1.36–2.11). Larger effect size was found in our study (OR = 2.74, 95% CI, 2.33–3.23), which might be attributed to a synergistic effect between LBP and sleep. These findings suggest that sleep duration plays an essential role in depressive symptoms. It is gratifying to see that behavioral interventions can extend sleep duration (46). Further prospective studies are necessary to evaluate the psychological effects of such interventions among people with LBP.

SRH reflects respondents' overall subjective perception of their health status. Although it is a single-item measure, it has been endorsed as a valid predictor of future health outcomes (47, 48). In this study, those with poor SRH had a greater prevalence of depressive symptoms compared to those with good SRH. Similar findings were demonstrated in another study on LBP (17). Because of the effectiveness and ease of implementation, clinical practitioners should pay attention to the evaluation of SRH, which may provide valuable information for identifying depressive states.

Multiple pains were highly prevalent in elderly patients with LBP (49). For our study, almost all respondents (*n* = 5330, 92.2%) reported at least one additional location of pain. Several studies have identified a linear relationship between the number of pain sites and overall health, sleep quality, and functional status (50, 51). Also, multisite pain was significantly associated with depression in German (52) and Netherlands (53) surveys. The underlying mechanism for the co-occurrence of pain/multisite pain and depressive symptoms remains controversial. It could be explained by several mainstream arguments: (a) it might indicate persistent widespread pain will eventually lead to depressive symptoms (54); (b) multiple pains could be a somatic manifestation of depressive symptoms (55); (c) they shared common pathological basis and biological pathways (56, 57); (d) some mediating factors might be at play between the two (58). But, the study's cross-sectional design constrained us to further explore the direction of the effect between the two. This issue required further studies to shed more light on it.

For the present study, we assessed physical functional disability with BADL and IADL. This disability not only contributed to decreased quality of life for the patients but also affected their mental health. Consistent with previous reports (17, 59, 60), participants with BADL/IADL disability had a greater likelihood of comorbid depressive symptoms. Unexpectedly, the relative magnitude of the association we reported between BADL disability and depressive symptoms (OR = 1.70, 95% CI,1.47–1.98) was smaller than that between IADL disability and depressive symptoms (OR = 1.95, 95% CI,1.70–2.24). Li and colleagues (61) observed gender differences whereby males with IADL disorder had higher rates of depressive symptoms, and conversely, BADL disorder was more likely to be associated with the development of depressive symptoms in females. Thus, our results may be attributed to the fact that a gender-stratified analysis was not performed. Further, 42.9% of individuals with LBP in this sample had ADL disability, suggesting that it is necessary to provide physical rehabilitation and psychological guidance in health care for people with LBP in middle and old age.

The study's strengths are that we selected the sample from a national survey and integrated systematic factors into the model. Furthermore, a rigorous sampling technique and standardized quality control procedures made the results more robust. However, there were some limitations to this current analysis. First, the study adopted a cross-sectional design. Thus, the cause-effect relationship between LBP and depressive symptoms could not be inferred, and further prospective studies are needed to better elucidate possible predictors. Second, the primary outcome was based on self-report from interviews. The estimation of these variables may be subject to potential information bias. Third, during the interview, no detailed information was available about the intensity and duration of LBP, which could have provided additional context for the analysis. Finally, we acknowledged that different methods to identify LBP might limit the ability to make direct comparisons with other studies.

## Conclusions

In conclusion, this study revealed that persons with LBP had a high prevalence of depressive symptoms. As such, healthcare professionals should be encouraged to screen people with LBP for depressive symptoms. Extra attention should be paid to groups at risk, including females, younger persons, those from central and western regions, those with shorter sleep duration, persons with poorer self-perceived health, those with multisite pain, and those with ADL disability. The information reported in the current study will be helpful when developing mental health care strategies for individuals with LBP and may serve as a reference for future targeted public health campaigns.

## Data availability statement

Original datasets presented in the study are available from the China Health and Retirement Longitudinal Study (CHARLS) website: http://charls.pku.edu.cn/en/.

## Ethics statement

The studies involving human participants were reviewed and approved by Peking University's Institutional Review Board. The patients/participants provided their written informed consent to participate in this study.

## Author contributions

CH, LX, and QL designed the study and were responsible for data analysis and manuscript writing. JZ extracted and cleaned the data. XH, HC, and LG provided advice on the first draft and revised essential details. All participants read and approved the submitted version.

## Funding

This study was supported by Sichuan Province Science and Technology Support Program (2021YJ0016).

## Conflict of interest

The authors declare that the research was conducted in the absence of any commercial or financial relationships that could be construed as a potential conflict of interest.

## Publisher's note

All claims expressed in this article are solely those of the authors and do not necessarily represent those of their affiliated organizations, or those of the publisher, the editors and the reviewers. Any product that may be evaluated in this article, or claim that may be made by its manufacturer, is not guaranteed or endorsed by the publisher.
